# Laricitrin ameliorates lung cancer-mediated dendritic cell suppression by inhibiting signal transducer and activator of transcription 3

**DOI:** 10.18632/oncotarget.13240

**Published:** 2016-11-09

**Authors:** Wei-An Chang, Jen-Yu Hung, Shu-Fang Jian, Yi-Shiuan Lin, Cheng-Ying Wu, Ya-Ling Hsu, Po-Lin Kuo

**Affiliations:** ^1^ Graduate Institute of Clinical Medicine, College of Medicine, Kaohsiung Medical University, Kaohsiung, Taiwan; ^2^ Division of Pulmonary and Critical Care Medicine, Kaohsiung Medical University Hospital, Kaohsiung, Taiwan; ^3^ School of Medicine, College of Medicine, Kaohsiung Medical University, Kaohsiung, Taiwan; ^4^ Graduate Institute of Medicine, College of Medicine, Kaohsiung Medical University, Kaohsiung, Taiwan; ^5^ Institute of Medical Science and Technology, National Sun Yat-Sen University, Kaohsiung, Taiwan

**Keywords:** laricitrin, dendritic cell, lung cancer, IL-10, STAT3

## Abstract

Natural polyphenolic compounds of grapes and their seeds are thought to be therapeutic adjuvants in a variety of diseases, including cancer prevention. This study was carried out to investigate the effect of grape phenolic compounds on the regulation of cancer-mediated immune suppression. Laricitrin exhibits the greatest potential to ameliorate the suppressive effects of lung cancer on dendritic cells’ (DCs’) differentiation, maturation and function. Human lung cancer A549 and CL1-5 cells change the phenotype of DCs that express to high levels of IL-10 and prime T cells towards an immune suppression type-2 response (Th2). Laricitrin treatment stimulated DC differentiation and maturation in the condition media of cancer cells, a finding supported by monocyte marker CD14's disappearance and DC marker CD1a's upregulation. Laricitrin decreases expression of IL-10 in cancer-conditioned DCs, and subsequently switches CD4^+^ T cell response from Th2 to Th1 *in vitro* and *in vivo*. Reversal of laricitrin on lung cancer-induced DCs’ paralysis was via inhibiting the phosphorylation of signal transducer and activator of transcription 3 (STAT3). Laricitrin also potentiated the anticancer activity of cisplatin in mouse models. Thus, laricitrin could be an efficacious immunoadjuvant and have a synergistic effect when combined with chemotherapy.

## INTRODUCTION

Lung cancer is one of the leading causes of cancer mortality in the world [1]. In spite of aggressive treatment with surgery, radiation and chemotherapy, long-term survival rates remain low for lung cancer patients [2]. Immunotherapy is considered a potentially effective strategy when conventional managements have achieved maximum advantage or are no longer effective in eradicating cancer [3, 4]. However, cancer actively develops diverse mechanisms, such as producing immunosuppressive factor to attenuate antitumor immunity developed by the host, thereby limiting the success of immunotherapy [4]. Identification and overcoming of the multiple mechanisms of cancer-mediated immune suppression can provide a novel strategy for new lung cancer therapies.

Dendritic cells (DCs) play critical roles in initiating innate and adaptive immune responses, which induce, regulate and maintain T-cell immunity, and are essential for the induction of anticancer immune responses [5, 6]. Growing evidence has shown that the appearance of tolerogenic DCs may contribute to profoundly immune-suppressive tumor microenvironments (TME) [7]. DCs isolated from animals and human cancer patients have phenotypic aberrations and functional loss, including differentiation insufficiency, immature DCs accumulation and production of anti-inflammatory cytokines [8]. As a result, DCs fail to activate cancer-reactive T cells and/or skew cytokine production from type-1 response (Th1) towards a Th2 response [9, 10]. Dysfunctional DCs are found in patients with various cancers and in experimental mice with transplanted or spontaneous malignancies [11, 12]. Development of agents that improves cancer-mediated DCs dysfunction can provide new therapies for cancer treatment.

Natural bioactive phytochemicals have been widely used for the prevention and treatment of many diseases for centuries. By increasing the host's anti-tumor immunity, natural phytochemicals are considered potential alternatives for the development of more effective therapeutic or preventative strategies for various diseases [13, 14]. Active components of grapes have been reported to inhibit UV-induced immunosuppression, which is assumed to decrease the risk of photocarcinogenesis [15, 16]. Alterations in the expression of immunoregulatory cytokines interleukin (IL)-10 and IL-12, and DCs activation are responsible for the inhibitory effect of grape seed derivatives on UV-induced immunosuppression [16]. In addition, the bioactive component of grape seeds has also been found to potentiate anti-cancer activity of doxorubicin via an immunomodulatory mechanism [17]. Laricitrin, syringetin, resveratrol and piceatannol, are phenolic compounds found in red grapes and *Vaccinium uliginosum* L [18, 19]. This study demonstrates that laricitrin provides the highest level of efficacy to improve lung cancer-mediated DC suppression through the down-regulation of the STAT3/IL-10 signaling pathway. Moreover, laricitrin potentiates the anti-cancer activity of cisplatin *in vivo*. These results suggest that laricitrin acts as an effective adjuvant immune-based agent for cancer treatment by impeding DCs’ function.

## RESULTS

### Laricitrin restores differentiation and maturation of DCs

**Figure 1:**
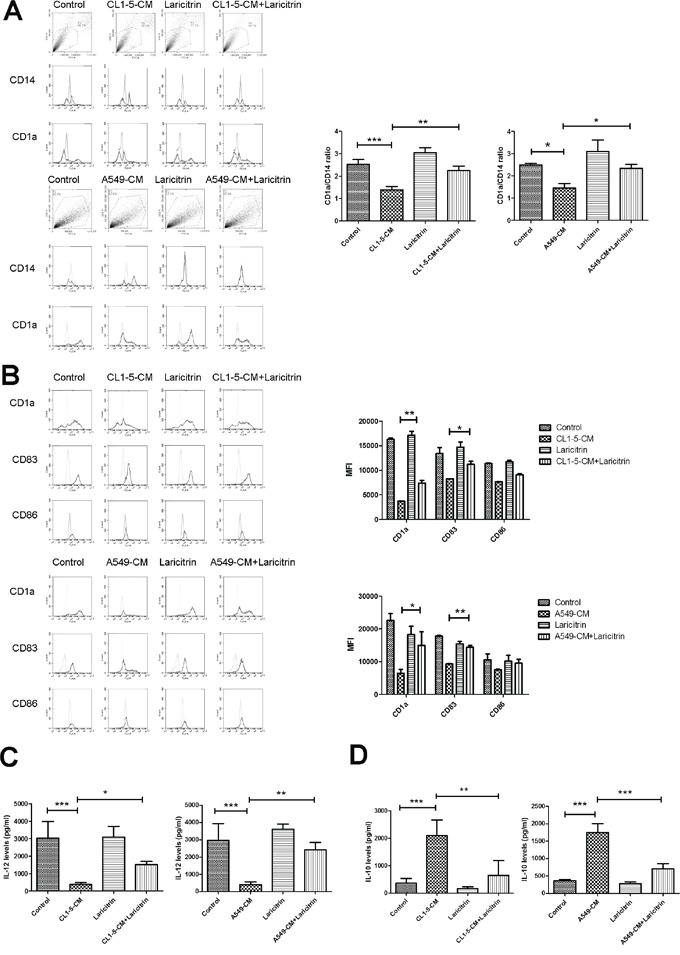
The effect of laricitrin on DCs’ differentiation and maturation Laricitrin improved the inhibitory effects of lung cancer on DCs’ differentiation **A.** and maturation **B.** Laricitrin decreased the effect of lung cancer on IL-12 downregulation **C.** and IL-10 upregulation **D.** CD14^+^ monocytes were cultured in RPMI 1640 medium containing GM-CSF (20 ng/ml) and IL-4 (10 ng/ml), with or without A549-CM or CL1-5-CM (20%), for 5 days. For DCs’ maturation, cells were primed by IFN-γ for 2 h, then activated by LPS (100 ng/ml) for another 2 days. Laricitrin-treated DCs were generated by same procedure but contained laricitrin (2 μM). The expression of various surface markers was assessed by flow cytometry. The levels of cytokines were determined using Magnetic Luminex Performance Assay kits. All results are representative of at least three independent experiments, and each value is the mean ± SD of three determinations. *p<0.05, **p<0.01, ***p<0.001.

To investigate whether the phenolic compounds of grapes improve tumor immunity evasion, we assessed the influence of laricitrin, syringetin, resveratrol and piceatannol on impairment of lung cancer on DCs’ differentiation and function. We assessed the effects of these phenolic compounds in CL1-5 and A549 human lung adenocarcinoma that is the most comment of lung cancer. Previously our study showed that lung cancer cells impair the differentiation and function of DCs, which is supported by changing phenotype (decreasing CD1a up-regulation and the appearance of CD14) and expression of high levels of IL-10 [20]. Therefore, IL-10 expression and CD1a/CD14 ratio are critical indicators for assessing the degrees of differentiation and function of DCs in lung cancer. We first assessed the effect of laricitrin, syringetin and piceatannol on the expression of IL-10. The result shows that lung cancer A549 and CL1-5 cells stimulated DCs to express a large amount of IL-10. Laricitrin, syringetin, resveratrol, and piceatannol decreased the expression of IL-10 in both A549 and CL1-5-conditioned DCs at the concentration without cytotoxicity for both DCs and cancer cells (Table 1 and Supplementary Figure 1). Laricitrin had the greatest activity, therefore, in a later study we will focus on laricitrin. Figure 1A shows that A549 and CL1-5-CM impairs the differentiation of DCs from CD14^+^ monocytes when compared to control-CM. However, the impairment of A549 and CL1-5 cells on DCs differentiation was reversed by laricitrin treatment.

**Table 1:** The effect of grape phenolic compounds on the expression of IL-10 in DCs without LPS stimulation

	IL-10 levels (pg/ml)			
	Control-CM	CL1-5-CM	Control-CM	A549-CM
Control	58.55 ± 11. 5	229.50 ±16.97^*^	57.95 ± 10.91	211.77 ± 19.43^*^
Laricitrin	59.99 ± 13.1	55.72 ± 7.14^#^	55.26 ± 9.01	70.74 ± 14.71^§^
Syringetin	41.50 ± 5.42	161.93 ± 3.15^#^	56.65 ± 13.28	168.22 ± 8.23^§^
Piceatannol	50.04 ±7.67	170.40 ± 6.22^#^	46.18 ± 5.21	157.95 ± 9.87^§^
Resveratrol	67.11± 3.99	85.02 ± 12.89^#^	53.86 ± 6.79	147.56±12.82^§^

All results are representative of at least three independent experiments, and each value is the mean ± SD of three determinations.

* p<0.05 vs. control-CM,

# p<0.05 vs. CL1-5-CM

§ p<0.05 vs. A549-CM.

We next assessed the effect of laricitrin on lung cancer-mediated reduction of the maturation of DCs. Exposure of DCs to A549 and CL1-5-CM decreased all mature markers of DCs after LPS stimulation, including CD1a, CD83, and CD86. Laricitrin prevented the reduction of lung cancer cells’ effect on DCs’ maturation (Figure 1B). In addition, A549- and CL1-5-CM also decreased the ability of DCs to produce proinflammatory cytokine IL-12, but increased secretion of immunosuppressive IL-10 after LPS stimulation. Laricitrin also prevented the effects of lung cancer cells on IL-12 inhibition and IL-10 induction in DCs (Figure 1C and 1D).

### Laricitrin restores DCs function on T cell activation

**Figure 2:**
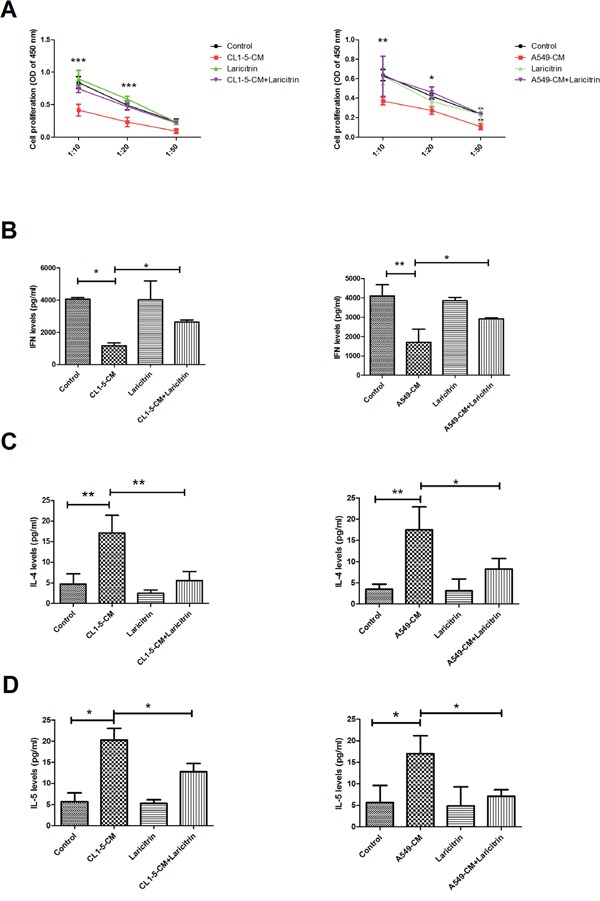
The effects of laricitrin on the function of DCs **A.** Laricitrin restored activation of DCs on T cell proliferation. Laricitrin changes the expression of IFN-γ **B.**, IL-4 **C.**, and IL-5 **D.** on CD4 T cell stimulated by DCs or lung cancer-conditioned DCs. Various types of DCs described in Figure 1 were incubated with allogeneic naive CD4^+^ T cells at DCs/T cell ratios of 1:10, 1:20, and 1:50. Cell proliferation of T cells was measured by BrdU incorporation. The expression of cytokines (DCs: T cell =1:20) was assessed by Magnetic Luminex Performance Assay kits. All results are representative of at least three independent experiments, and each value is the mean ± SD of three determinations. *p<0.05, **p<0.01, ***p<0.001.

The major function of DCs is to present antigens to the T cells and priming T response [7]. In comparison with those cells cultured in control-CM, A549 and CL1-5-CM–conditioned DCs showed impaired ability to induce naive CD4^+^ T cell proliferation (Figure 2A). The effect of DCs on T cell proliferation was restored by laricitrin treatment, however. Significantly, co-culture of naive CD4^+^ T cells with A549 and CL1-5-CM-conditioned DCs led to secretion of significantly lower amounts of Th1 cytokines (IFN-γ) and increased Th2 cytokine IL-4 and IL-5 production, when compared with CD4^+^ T cells stimulated by control-CM-conditioned DCs. The expression of Th1 cytokine IFN-γ, was also restored by laricitrin in CD4^+^ T cells after being co-cultured with A549 and CL1-5-CM–conditioned DCs (Figure 2B to 2D). In contrast, the production of Th2 cytokines IL-4 and IL-5 was also decreased by laricitrin in CD4^+^ T cells after being co-cultured with A549 and CL1-5-CM–conditioned DCs (Figure 2C and 2D).

### Laricitrin restores differentiation, maturation and function of DCs by decreasing IL-10 expression

**Figure 3:**
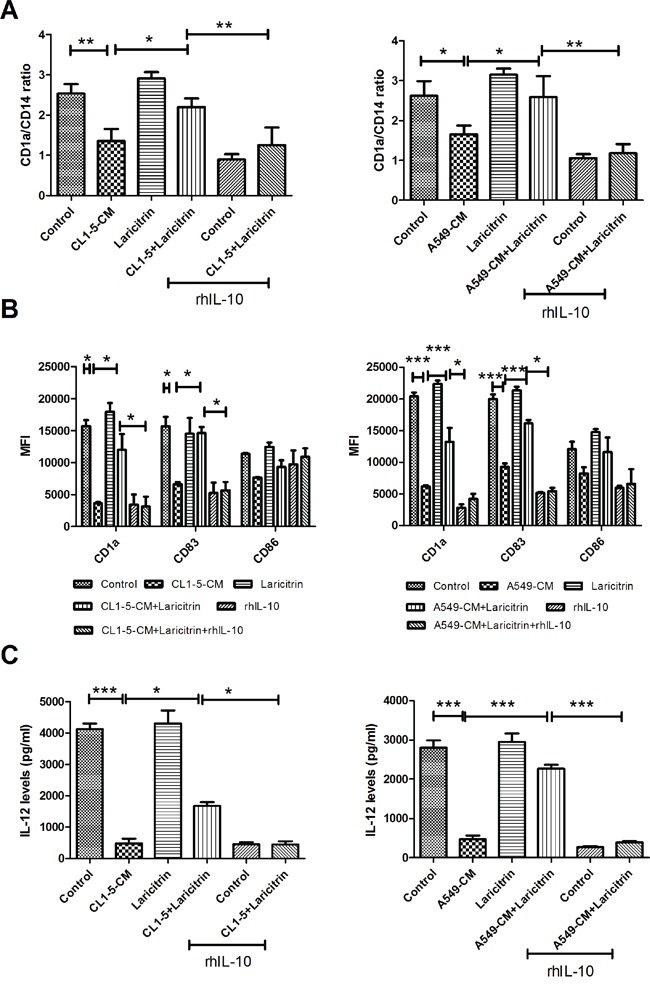
Laricitrin improves DCs’ differentiation and maturation by decreasing IL-10 expression in lung cancer-conditioned DCs The addition of rhIL-10 attenuated the effect of laricitrin on DCs’ differentiation **A.**, maturation **B.** and IL-12 downregulation **C.** under lung cancer condition media. Various types of DCs were generated as described above, with or without rhIL-10 (10 ng/ml). The expression of various surface markers was assessed by flow cytometry. The levels of cytokines were determined using Magnetic Luminex Performance Assay kits. All results are representative of at least three independent experiments, and each value is the mean ± SD of three determinations. *p<0.05, **p<0.01, ***p<0.001.

IL-10 has been reported to be a major factor inhibiting the differentiation of DCs from monocytes in cancer [21]. We consequently assessed whether the inhibitory effect of laricitrin on IL-10 production contributes to improved effects of laricitrin on DC differentiation and maturation. Laricitrin reversed the deleterious effects of A549- and CL1-5-CM on DC differentiation. Addition of recombinant human IL-10 (rhIL-10) attenuated the effect of laricitrin on the amelioration of DCs’ inadequate differentiation induced by lung cancer (Figure 3A). Similarly, rhIL-10 also decreased laricitrin activity on the amendment of DCs’ insufficient maturation and IL-12 downregulation induced by lung cancer (Figure 3B and 3C).

**Figure 4:**
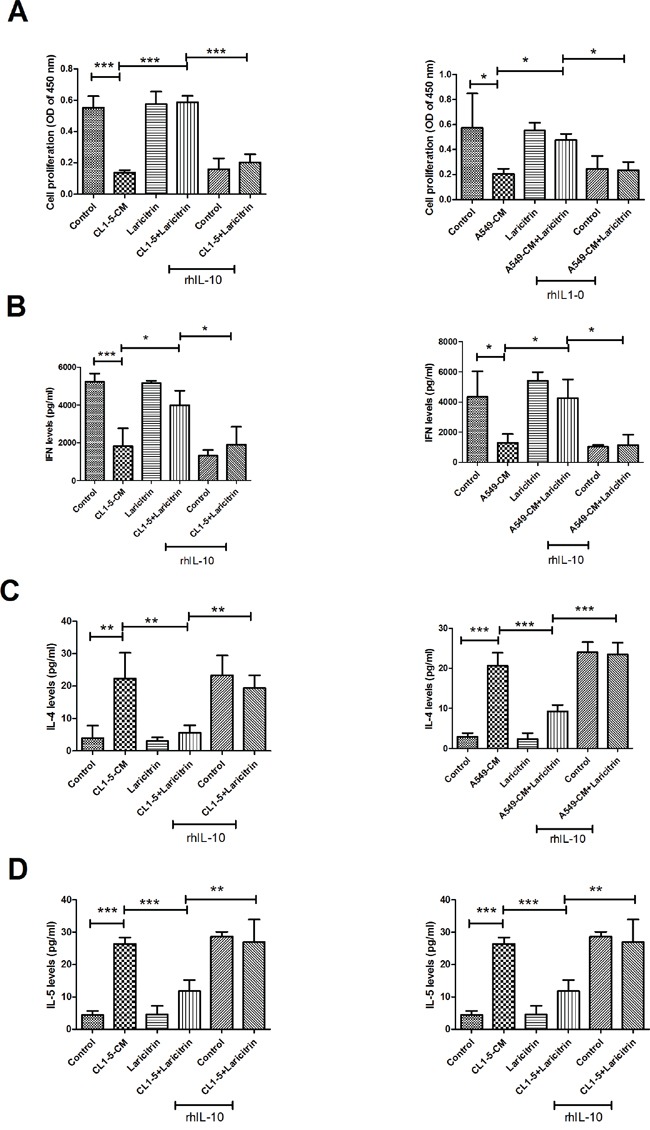
Laricitrin ameliorated DC function by decreasing IL-10 expression in lung cancer-conditioned DCs The addition of rhIL-10 attenuated the reverse effect of laricitrin on proliferation **A.**, IFN-γ **B.**, IL-4 **C.** and IL-5 **D.** production in CD4 T cell stimulated by DC or lung cancer conditioned DC. Various types of DCs were generated as described above, with or without IL-10 (10 ng/ml), and then incubated with allogeneic naive CD4^+^ T cells (DCs: T cell =1:20). Proliferation of T cells was measured by BrdU incorporation. The levels of cytokines were determined using Magnetic Luminex Performance Assay kits. All results are representative of at least three independent experiments, and each value is the mean ± SD of three determinations. *p<0.05, **p<0.01, ***p<0.001.

We next evaluated whether IL-10 contributes to the influence of laricitrin on restored T cell priming function of DCs. Addition of rhIL-10 effectively decreased the stimulation of laricitrin on T cell proliferation, which was cultured with A549 and CL1-5-CM-conditioned DCs (Figure 4A). In addition, the effects of laricitrin on Th1 cytokines (IFN-γ) upregulation and Th2 cytokine IL-4 and IL-5 downregulation in T cells were also inhibited by adding rhIL-10 (Figure 4B to 4D).

### Laricitrin inhibits IL-10 expression by decreasing STAT3 activation

**Figure 5:**
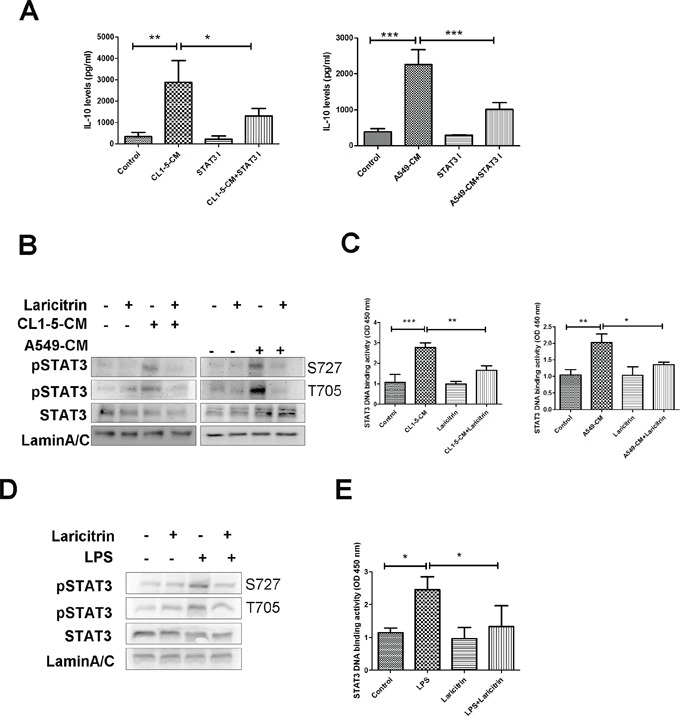
Laricitrin decreased IL-10 expression by inhibiting STAT3 **A.** STAT3 inhibitor decreased lung cancer-mediated IL-10 expression. Laricitrin inhibited lung cancer-mediated upregulation on STAT3 activation **B.** and DNA binding activity **C.** in monocytes. Laricitrin inhibited LPS-mediated upregulation on STAT3 activation **D.** and DNA binding activity **E.** in monocytes. CD14^+^ monocytes were pretreated with laricitrin (2 μM) for 1 h, and then A549, CL1-5-CM or LPS (100 ng/ml) added for 30 min. The phosphorylation of STAT3 was assessed by immunoblot. The DNA binding activity of STAT3 was assessed by EMSA analysis. All results are representative of at least three independent experiments, and each value is the mean ± SD of three determinations. *p<0.05, **p<0.01, ***p<0.001.

STAT3 has been reported to regulate the expression of IL-10 in various immune cells [22, 23]. We assessed whether STAT3 is responsible for IL-10 upregulation in lung cancer-conditioned DCs and if it is a target for laricitrin. First, we evaluated the role of STAT3 on IL-10 upregulation using STAT3 inhibitor. Pretreatment of CD14^+^ monocytes with STAT3 inhibitor decreased A549- and CL1-5-CM-induced IL-10 expression in CD14^+^ monocytes (Figure 5A), suggesting that STAT3 is a critical regulator in IL-10 expression. Next, we assessed the effect of laricitrin on STAT3 activation and activity. Both A549- and CL1-5-CM increased the phosphorylation of STAT3, and laricitrin decreased the lung cancer-induced activation of STAT3 in CD14^+^ monocytes (Figure 5B). EMSA analysis also revealed that A549- and CL1-5-CM increased the DNA binding activity of STAT3, and laricitrin reduced the lung cancer-induced enhancement of STAT3 on DNA binding capacity in CD14^+^ monocytes (Figure 5C). Moreover, laricitrin also inhibited LPS-mediated STAT3 phosphorylation and activity enhancement in CD14^+^ monocytes (Figure 5D and 5E), suggesting that laricitrin may be a STAT3 inhibitor.

### Laricitrin potentiates the chemotherapeutic effect of cisplatin by improving anti-cancer immunity in mice

**Figure 6:**
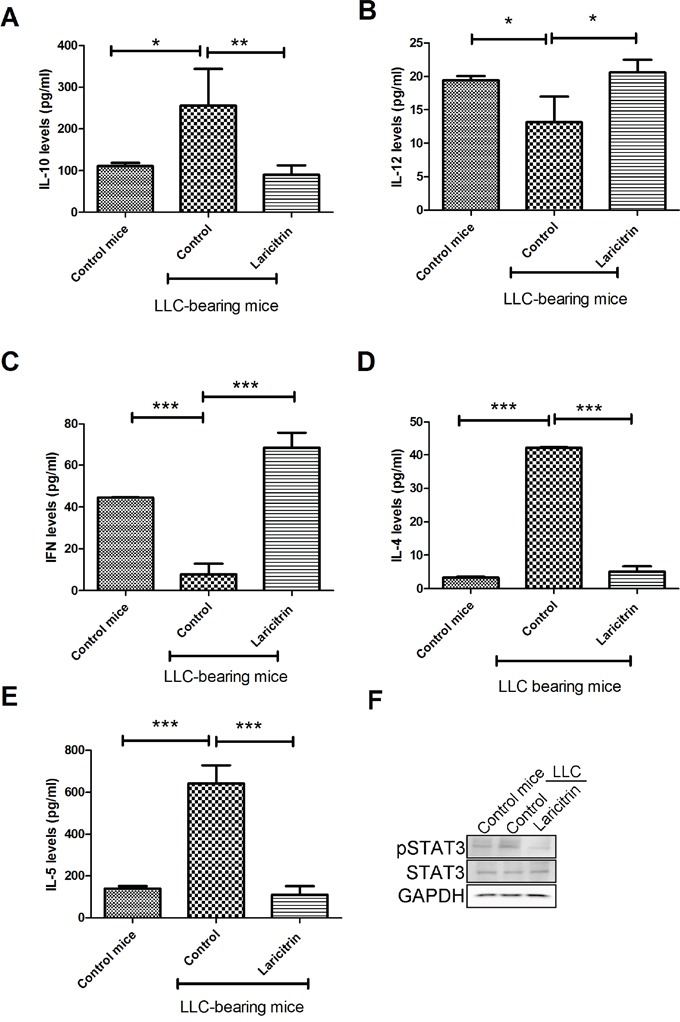
Laricitrin improves anticancer immunity *in vivo* Laricitrin decreases IL-10 **A.** levels and increases IL-12 **B.** in DCs of the lungs of mice with LLC transplantation. Laricitrin changes IFN-γ **C.**, IL-4 **D.** and IL-5 **E.** and expression in CD4^+^ T cells in mice with or without LLC transplantation. **F.** Laricitrin decreases the activation of STAT3 in DCs. LLC cells were implanted into C57BL/6 mice via tail vein injection. The mice were then randomly divided into two groups: the laciritin-treated group was given i.p. injection daily with laricitrin (dose: 30 mg/kg of body weight) while the control group was given an equal volume of normal saline. Tumor-bearing mice were euthanized 14 days after transplantation. DC (CD11c^+^, F4/80^-^) and CD4 T cells were isolated from the lungs of mice. The expression of cytokines was assessed by Magnetic Luminex Performance Assay kits. The results are reported as mean ± SD; *p<0.05, **p<0.01, ***p<0.001.

To investigate whether laricitrin could alter immuno-surveillance in mice, LLC were transplanted into mice by tail vein injection. The mice were euthanized after 14 days, and DC (CD11c^+^F4/80^-^) and CD4^+^ T cells from the tumor nodules of the lungs were isolated and examined. IL-10 expression in DCs (CD11c^+^/F4/80^-^) of the lungs of mice with LLC transplantation was higher than the DCs isolated from the lungs of normal mice. Administration of laricitrin decreased IL-10 levels and increased IL-12 in DCs of the lungs of mice with LLC transplantation (Figure 6A and 6B). Laricitrin treatment also switched Th2 response (IL-4 and IL-5 secretion) toward Th1 response (IFN production) in CD4^+^ T cells, when compared to vehicle-treated mice with LLC transplantation (Figure 6C to 6E). Moreover, laricitrin also decreased the activation of STAT3 in DCs of the lungs of mice with LLC transplantation (Figure 6F).

**Figure 7:**
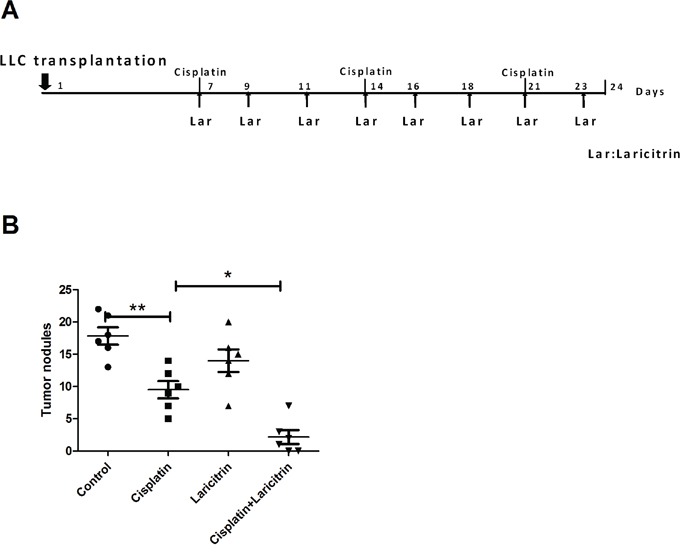
Laricitrin potentiated the anticancer activity of cisplatin *in vivo* **A.** Flow chart representation of *in vivo* experimental design and treatment schedule. **B.** The tumor nodules of lungs of mice. Tumor-bearing mice were euthanized, their lungs removed, and the tumor nodules counted. The results are reported as mean ± SD; *p<0.05, **p<0.01.

Since the combination of chemotherapy and immunotherapy is considered synergistic and enhances the clinical response, we evaluated whether laricitrin could synergize with cisplatin, a commonly used drug for treating lung cancer, to produce more powerful antitumor effects. LLC were transplanted into the lungs of mice by tail vein injection. After 1 week, we treated LLC tumor-bearing mice with i.p. injections of cisplatin (2.5 mg/kg/week) with or without three doses of laricitrin (30 mg/kg) within 5 days (Figure 7A). Combined laricitrin treatment significantly decreased tumor nodules in the lungs of mice, compared to the group treated with cisplatin (Figure 7B).

## DISCUSSION

The tumor microenvironment is widely considered immunosuppressive, resulting in immune *evasion* and tumor progression [24, 25]. This study is the first to demonstrate that laricitrin improves lung cancer-induced immune inhibition by restoring DCs’ differentiation, maturation and function, resulting in the recovery of anticancer immunity (Figures 1 to 2). Moreover, laricitrin also potentiates the anticancer activity of cisplatin in a mouse model (Figure 7). These findings suggest that a combination of laricitrin and cisplatin represents a novel approach to chemoimmunotherapy.

IL-10 has been reported to be found at high levels in a variety of human malignancies, including lung cancer [26, 27]. IL-10 has the ability to prevent the differentiation of DCs from monocytes, as well as impairing the potent APC function of DCs. In addition, IL-10 impedes the ability of DCs to stimulate T cells [28]. The presence of IL-10-producing DCs within tumors is associated with cancer antigen-specific immune responses and increased Treg populations, which have been implicated in playing a crucial role in the occurrence of tumor-mediated immune evasion [29]. Neutralizing IL-10 by anti-IL-10R mAbs significantly improves the anti-tumor immune response in certain animal models of cancer [29]. Our results found that laricitrin decreases the expression of IL-10 in DCs (Table 1, Figures 1 and 6), restoring the DCs’ differentiation, maturation (Figure 1) and function in TME. In addition, laricitrin treatment also increased tumor-destructive Th1 response by upregulation of the IL-12/IL-10 ratio in DCs in the tumor microenvironment (Figures 1 and 6). These results suggest that laricitrin could be an effective adjuvant to enhance anticancer immunity of hosts with malignancies.

STAT3 transcription factors are a point of convergence of several most critical oncogenic signaling and upstream modulators of diverse tumor-promoting factors [30, 31]. The STAT3 family plays an important role in determining the differentiation of cell lineages. STAT3 is also thought to be an important mediator of tumor immune suppression [32]. Overactivation of STAT3 not only decreases the differentiation and maturation of DCs, but also promotes expression of immunosuppressive factors such as IL-10 and VEGF, and inhibits production of various Th1 immunostimulatory molecules [32, 33]. However, inhibition of STAT3 elicits multicomponent antitumor immunity [34]. This study investigates how lung cancer increases the activation and DNA binding activity of STAT3, which in turn enhances the expression of IL-10 in DCs. Laricitrin decreases the lung cancer-mediated activation of STAT3, subsequently reducing IL-10 levels in DCs (Figures 5 and 6). According to the studies presented here, laricitrin may have a novel mechanism for inhibiting STAT3 activation in DCs, resulting in enhancement of anticancer immunity by restoring DC function and Th1 response in cancer niches.

Growing evidence suggests that the greatest potency and specificity of anticancer response can be achieved by a combination of conventional therapy with immunotherapy, which is more efficacious than either of these two treatments alone [35, 36]. Therapeutics change immunomodulatory molecules to enhance antitumor immunity, which can be sufficient to eradicate the malignancy [36]. A neutral polysaccharide fraction of *Panax ginseng* potentiates the effect of 5-fluorouracil in sarcoma-180 tumor-bearing mice by increasing natural killer cells’ cytotoxicity and macrophage function [37]. Induction of immunogenic tumor cell death can amplify cisplatin's therapeutic efficacy [38]. Combined treatment of cisplatin and anti-CD137 or anti-PD-1 monoclonal antibodies (mAbs) also creates a synergistic therapeutic effect in an ID8 mouse ovarian cancer model [39]. In this study, we found that laricitrin not only enhances DCs’ function, increasing IL-12 and decreasing IL-10 expression, but also switches tumor-promoting Th2 to a tumor-destructive Th1 response *in vivo* (Figure 6). Furthermore, laricitrin significantly enhances the efficacy of cisplatin against lung cancer in the mouse model (Figure 7). Our results provide a novel therapeutic strategy that appears to hold great promise for lung cancer patients.

**Figure 8:**
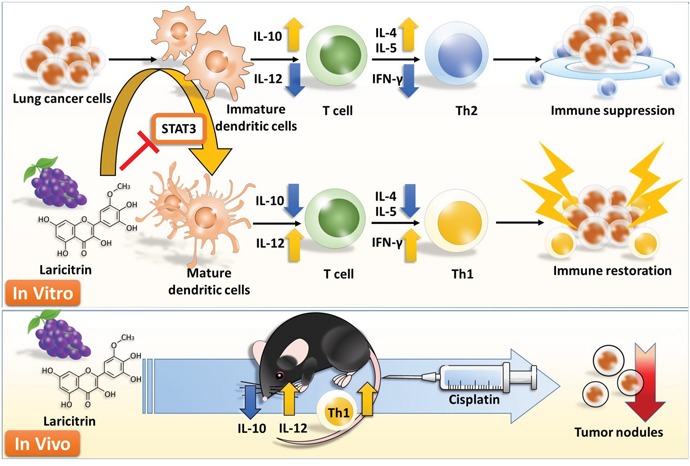
Schematic diagram proposing how laricitrin impairs lung cancer-mediated immune suppression

In conclusion, laricitrin protects against lung cancer-mediated immune suppression by directly improving insufficient DC differentiation and maturation mediated lung cancer, and by restoring the Th1 response of T cells. In addition, laricitrin also increases the anticancer efficacy of cisplatin against lung cancer, suggesting that laricitrin may serve as a future auxiliary immunotherapeutic agent to strengthen chemotherapy in fighting lung cancer (Figure 8).

## MATERIALS AND METHODS

### Reagents

Laricitrin and syringetin were purchased from Extrasynthese (Genay, France) (Purity >99.3% by HPLC analysis); Piceatannol, resveratrol, lipopolisaccharide (LPS) and cisplatin were obtained from Sigma Chemical (St. Louis, MO). Recombinant Human (rh) IL-10 protein was obtained from R&D Systems (Minneapolis, MN).

### Cell culture

Lung cancer A549 and Lewis lung carcinoma (LLC) cells were purchased from American Type Culture Collection (ATCC) (Manassas, VA, USA). CL1-5 human lung adenocarcinoma cell line was generously provided by Dr. Pan-Chyr Yang (Department of Internal Medicine, National Taiwan University Hospital). The A549 cells were cultured in F-12K Medium (Kaighn's Modification of Ham's F-12 Medium) with 10% fetal bovine serum (FBS). CL1-5 and LLC were cultured in RPMI 1640 supplemented with 10% FBS and 1% penicillin-streptomycin (Gibco BRL, Life Technologies). For the collection of conditioned medium (CM), the CL1-5 and A549 cells (1×10^6^) were seeded in a 10 cm dish and the supernatants collected after a 24 h culture, filtered (0.22 mm), and designated as CL1-5-CM and A549-CM. All CMs were frozen and stored at -80°C, and used after a single thawing for study. The cell viability of CL1-5 and A549 was assessed by Premixed WST-1 Cell Proliferation Assay (Takara Bio Inc., Shiga, Japan).

### DCs generation and allogeneic mixed lymphocyte reaction (MLR)

Peripheral blood mononuclear cells (PBMCs), were purified from the blood of healthy, consenting donors by the Ficoll-Hypaque gradient (GE Healthcare Bio-Sciences, Little Chalfont, UK). The hospital's Institutional Review Board approved the study, and all donors provided informed consent in accordance with the Declaration of Helsinki. To avoid contamination with unwanted cells which interfere with monocyte differentiation and T cell activation, and to increase the purity of the selected cell population, we used positive selection system for isolation of monocytes and CD4 T cells. CD14^+^ monocytes and CD4^+^ T cells were isolated using CD14 and CD4 monoclonal antibody-conjugated magnetic beads (MACS MicroBeads, Miltenyi Biotec) from PBMC, according to the manufacturer's protocol. DCs were generated by culturing CD14^+^ monocytes in RPMI 1640 medium containing 10% FBS (Invitrogen, Carlsbad, CA) and 20 ng/mL GM-CSF and 10 ng/mL IL-4 (R&D Systems, Minneapolis, MN) with or without laricitrin for 5 days. The medium was replaced with fresh medium containing GM-CSF and IL-4 on day 3. A549 and CL1-5 conditioned DCs were generated by culturing CD14^+^ monocytes in RPMI 1640 medium containing FBS, GM-CSF, and IL-4 presenting in A549-CM (20%), or CL1-5-CM (20%), with or without laricitrin (2 μM), for 5 days. For maturation analysis, DCs were stimulated with LPS (100 ng/ml) for 2 days after priming with interferon-γ (IFN-γ) for 2 h [20].

MLR was carried out by culturing naive CD4^+^ T cells for a set number of mature DCs in 96-well plates for 4 days. T cell proliferation was assessed by BrdU Cell Proliferation Kit (Millipore), as described in a previous study [20]. The supernatants of T cells were collected after 1 day co-culture and then the cytokines were assessed.

### Flow cytometry analysis

DCs were stained by antibodies against human CD14 labeled by FITC, CD1a labeled by phycoerythrin (PE), CD83 labeled allophycocyanin (APC) and CD86 labeled PerCP-Cy™5.5 (BD Biosciences, San Jose, CA). The expression of each molecule was analyzed using an Acuri C6 flow cytometer (BD Biosciences).

### Measurement of secreted factors

Supernatants from DCs and T cells were collected. CD11c^+^F4/80- and CD4^+^ T cells were isolated from mice and cultured in RPMI1640 medium. After 24 h, the supernatants were harvested. The levels of various cytokines were quantified using human and mouse Magnetic Luminex Performance Assay kits (R&D Systems).

### Immunoblot analysis

CD14^+^ monocytes (4×10^6^) were pre-treated with laricitrin for 1 h, then LPS, A549- and CL1-5-CMs were added for 30 min. The nuclear extracts were prepared using *nuclear extract kit* (Active Motif^®^, Carlsbad, CA, USA). Equivalent amounts of protein were resolved by the SDS-PAGE and transferred to PVDF membranes. After the membranes were blocked in Tris-buffer saline containing 0.05% Tween 20 (TBST) and 5% non-fat powdered milk, they were incubated overnight with primary antibodies against p-STAT3 (Tyr705 and Ser727, Cat no. 4093 and 9134, 1:1000; Cell Signaling Technology) and Lamin A/C (Cat no. 612162, 1:1000; BD Biosciences) at 4°C. After washing three times with TBST, the membranes were incubated with horseradish peroxidase-labeled secondary antibody for 1 h and then re-washed. Detection was performed using an enhanced chemiluminescence blotting detection system (Millipore).

### Electrophoretic mobility shift assay (EMSA)

The DNA binding activity of STAT3 transcription factor was examined using EMSA-based ELISA kits (Trans-AM STAT3 kit, Active Motif^®^, Carlsbad, CA, USA). The nuclear fractions obtained were determined by using *nuclear extract kit* according to the manufacturer's protocol. Nuclear protein extract (5 μg) was added to each well of a 96-well plate coated binding sequence (5′-TTCCCGGAA-3′). After binding, antibodies against STAT3 were added, followed by the HRP-conjugated secondary antibodies and a substrate solution. The DNA binding activity of STAT3 was calculated by averaging the duplicate absorbance (450 nm) for each sample and subtracting the mean of blank (oligonucleotide only) absorbance.

### Animal model

Male C57BL/6 mice from the National Science Council Animal Center (Taipei, Taiwan) were maintained in pathogen-free conditions. LLC cells were implanted into C57BL/6 mice via tail vein injection. The mice were then randomly divided into two groups: the laricitrin-treated group was given intra-peritoneal (i.p.) injection daily with 0.2 ml of laricitrin (dose: 30 mg/kg of body weight) while the control group was given an equal volume of normal saline. The dose of laricitrin was estimated to be equivalent to the pharmacokinetics profile of grape phenols [40]. Tumor-bearing mice were euthanized 14 days after transplantation. Lungs of LLC bearing mice were collected and minced. Single cell suspensions were obtained after enzymatic digestion (1 mg/ml collagenase A; Roche Diagnostics) and 100 IU/ml type I DNase (Sigma-Aldrich) for 2 h at 37°C and 5% CO_2_ in RPMI 1640 medium. Single cell suspension was filtered through a 70 μm nylon mesh (BD Biosciences), and cells were washed twice by PBS. CD11c^+^ cells were purified using anti-CD11c monoclonal antibody-conjugated magnetic beads (MACS MicroBeads, Miltenyi Biotec). F4/80^+^ cells were depleted from CD11c^+^ cells by using F4/80^+^ antibody-biotin beads (Miltenyi Biotec). CD4^+^ T cells were isolated from lungs using anti-CD4 monoclonal antibody-conjugated magnetic beads (MACS MicroBeads, Miltenyi Biotec).

To study the efficacy of the combination of laricitrin and cisplatin, mice underwent LLC transplantation (1×10^6^ cells/mouse) and were then randomly divided into four groups after 1 week: the laricitrin and cisplatin only-treated group was given intra-peritoneal (i.p.) injections with 0.2 ml of laricitrin (dose: 30 mg/kg of body weight, 3 times/week) or cisplatin (2.5 mg/kg/week), respectively; the laricitrin and cisplatin combination group was given i.p. injections with 0.2 ml laricitrin (dose: 30 mg/kg of body weight, 3 times/5 days) and cisplatin (2.5 mg/kg/week), whereas the control group was given an equal volume of normal saline. After 2.5-cycle treatment, the *lungs* of *mice* were removed and the tumor nodules counted. All animal experiments were performed according to a protocol approved by the Institutional Animal Care and Use Committee of Kaohsiung Medical University Hospital.

### Statistical analysis

Data were expressed as means ± SD. Multiple comparisons were evaluated by a one-way ANOVA, and differences in the mean values among groups were conducted by a Turkey post hoc analysis. *p* values < 0.05 were considered to be statistically significant.

## SUPPLEMENTARY MATERIALS FIGURE


