# Supplementary material for: Laricitrin ameliorates lung cancer-mediated dendritic cell suppression by inhibiting signal transducer and activator of transcription 3
Source: Oncotarget. 2016 Nov 9;7(51):85220–34. doi: 10.18632/oncotarget.13240 (PMC5356731; doi:10.18632/oncotarget.13240)
Supplement: Supplementary file 1 [file oncotarget-07-85220-s001.pdf]

Laricitrin ameliorates lung cancer-mediated dendritic cell suppression by inhibiting signal transducer and activator of transcription 3

SUPPLEMENTARY FIGURE

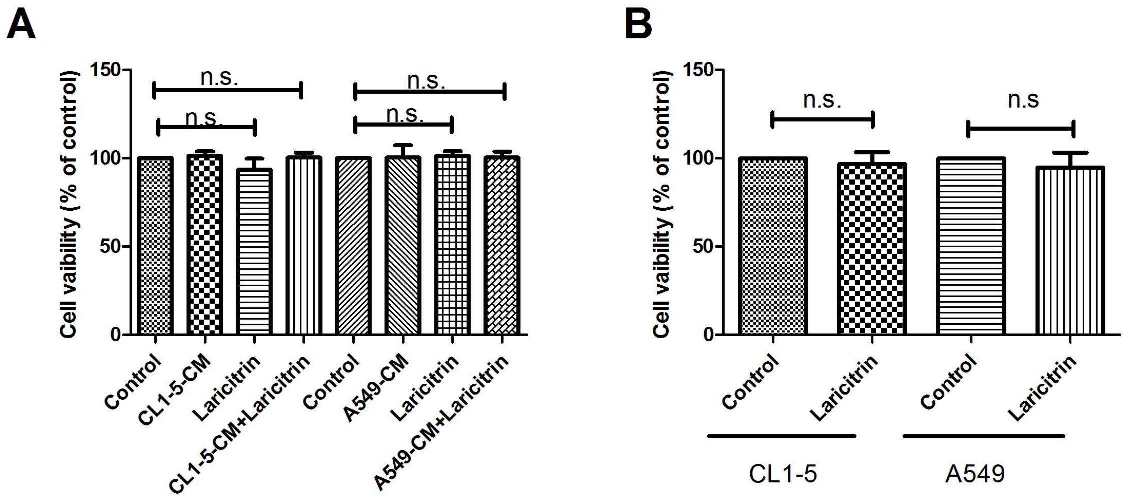

Supplementary Figure 1: Laricitrin did not affect the cell viability in DCs and lung cancer cells. The effect of laricitrin (2 μM) in the cell viability of DCs **A.** and lung cancer CL1-5 and A549 cells **B.** DCs were generated as described as Figure 1 legend. The cell viability was assessed by WST-1 after 5 day (DCs) and 48 h (lung cancer cells) treatment. The results are reported as mean ± SD; n.s, no significant ($p>0.05$).
